# Double outlet right ventricle

**DOI:** 10.3389/fped.2023.1244558

**Published:** 2023-09-25

**Authors:** Yolandee Bell-Cheddar, William A. Devine, Carlos-Eduardo Diaz-Castrillon, Laura Seese, Mario Castro-Medina, Raymond Morales, Christopher W. Follansbee, Tarek Alsaied, Jiuann-Huey I. Lin

**Affiliations:** ^1^ Division of Pediatric Cardiac Critical Care, UPMC Children's Hospital of Pittsburgh , Pittsburgh, PA, United States; ^2^ Department of Critical Care Medicine, University of Pittsburgh School of Medicine, Pittsburgh, PA, United States; ^3^ Department of Developmental Biology, University of Pittsburgh School of Medicine, Pittsburgh, PA, United States; ^4^ Department of Pediatric Cardiothoracic Surgery, UPMC Children's Hospital of Pittsburgh, Pittsburgh, PA, United States; ^5^ Division of Pediatric Cardiac Critical Care, Children's Hospital of New Orleans, New Orleans, LA, United States; ^6^ Division of Pediatric Cardiology, UPMC Children’s Hospital of Pittsburgh, Pittsburgh, PA, United States

**Keywords:** double outlet right ventricle, atrioventricular septal defect, ventricular septal defect, preoperative management, surgical management, genetics

## Abstract

This review article addresses the history, morphology, anatomy, medical management, and different surgical options for patients with double outlet right ventricle.

## Introduction

Double outlet right ventricle (DORV) was first described pathologically in 1957 (1). This pathoanatomical constellation was initially referred to as partial transposition of great arteries (TGA) with only the aorta transposed. Goor et al. gave the definition of DORV as the cardiac condition wherein both great arteries arise from the morphologic right ventricle (RV). Each great artery should have 50% or greater of the valve diameter coming from the morphologic RV, and there is almost always an interventricular communication: ventricular septal defect (VSD) or atrioventricular septal defect (AVSD) (2). However, in rare cases, interventricular communication may be absent. In addition, DORV is frequently associated with an assortment of other cardiac malformations. The consensus definition by the International Society for Nomenclature of Pediatric and Congenital Heart Disease (ISNPCHD) ultimately settled on “the hearts with both arterial trunks supported predominantly by underlying morphologic RV” where both great arteries arise entirely or predominantly from the morphological RV (3).

Yet, this definition may be too simplistic. In 1949, Taussig and Bing described this entity as a “complete” transposition and left position of the aorta, with subpulmonic VSD (4) (i.e., Taussig–Bing anomaly). Later in 1957, Witham classified DORV into three groups: those with pulmonary stenosis (PS), those without PS, and those with an AVSD as well as other malformations (1). However, only after 1972, Maurice Lev emphasized the importance of the position of the VSD, the great arteries, and the relationship between each other. This is the predominant classification that is most frequently used to classify this congenital anomaly in the modern era (5).

In essence, DORV “*is a purely descriptive term that is not used to name a discrete congenital cardiac malformation but rather to corral a broad range of phenotypes…*” (6). As such, no one picture should come to mind when one hears the term DORV.

## Cardiac morphology/pathology/embryological theories

**Figure 1:**
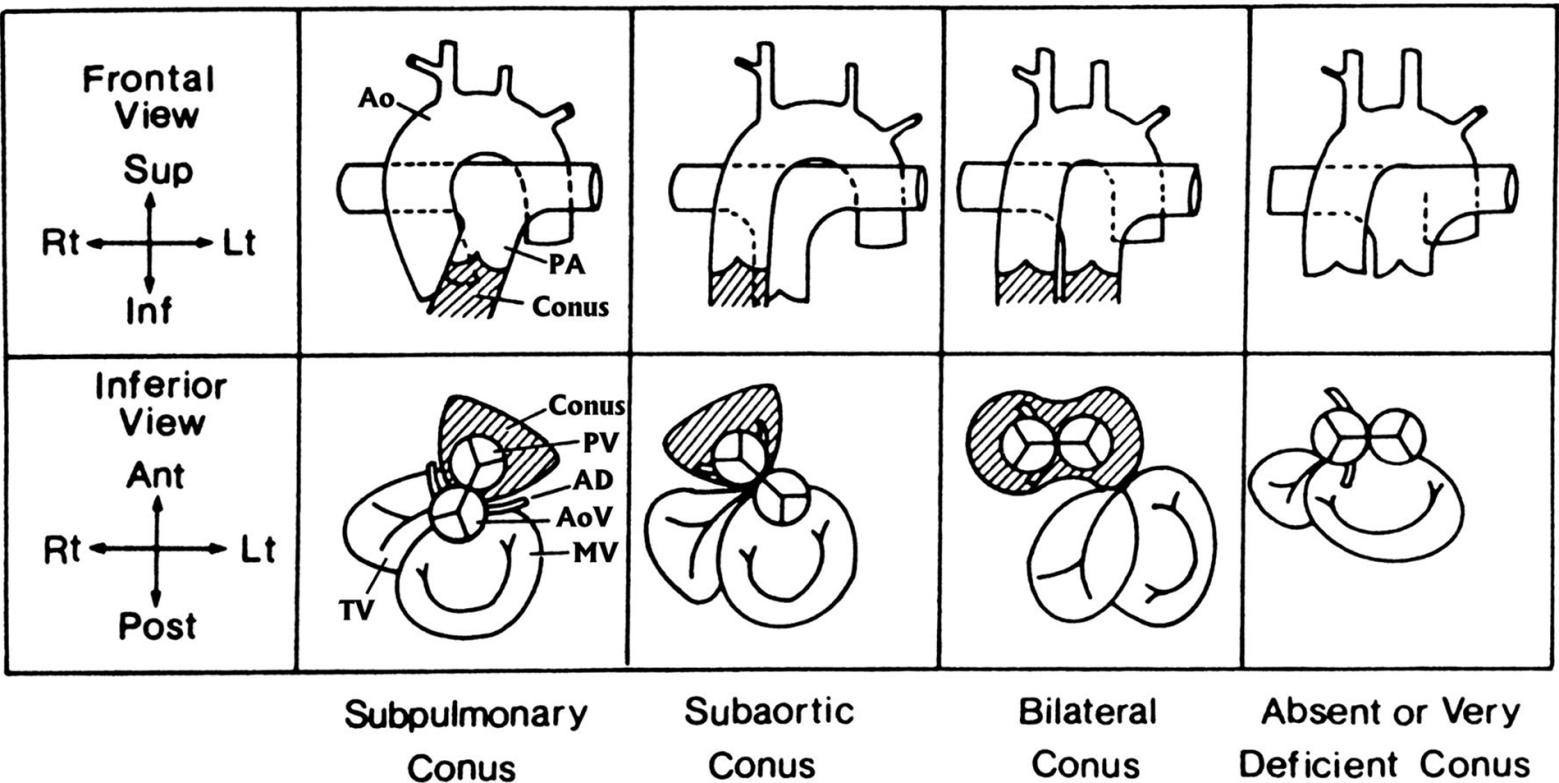
The four main anatomical types of conus arteriosus: subpulmonary, subaortic, bilateral, and absent or very deficient. The upper row shows the infundibulum (crosshatched) and great arteries as seen from the front (frontal view). The lower row shows the infundibulum (crosshatched); the semilunar valves—the aortic valve (AoV), indicated by the coronary arteries; the pulmonary valve (PV), indicated by the absence of coronary arteries; the atrioventricular valves; the bileaflet MV; and the trileaflet tricuspid valve (TV)—as seen from below (inferior view), similar to a subxiphoid two-dimensional echocardiogram. In all diagrams, a ventricular D-loop is assumed to be present. The subpulmonary conus is normal. Resorption of the subaortic conal free wall permits aortic-mitral fibrous continuity. A subpulmonary conus is associated with solitus normally related great arteries (diagrammed here), inversus normally related great arteries, and in the tetralogy of Fallot, both with solitus normally related great arteries and with inversus normally related great arteries. A subpulmonary conus can also be associated DORV with the hypoplastic left heart syndrome and with aortic-tricuspid fibrous continuity. The subaortic conus is characterized by resorption of the subpulmonary conal free wall, permitting pulmonary–mitral direct fibrous continuity. The presence of a complete muscular subaortic conus prevents aortic-atrioventricular fibrous continuity. The subaortic conus and great arteries shown here are associated with typical D-transposition of the great arteries. AD, anterior descending (coronary artery); Ant, anterior (ventral); Inf, inferior (caudad); Lt, left; Post, posterior (dorsal); Rt, right; Sup, superior (cephalad). Reproduced and modified with permission from Van Praagh and Van Praagh (10).

In anatomically normal hearts, D-looping of ventricles brings the left ventricle (LV) to the left with the aorta moving right and posterior while the right ventricle (RV) moves to the right with the pulmonary artery (PA) moving anterior and leftward. A muscular tissue called subpulmonary conus follows beneath. Normally, the subaortic conus resorbs and brings the aorta in continuity with the mitral valve (MV) or whichever atrioventricular (AV) valve is in that position. In DORV, the subaortic conus frequently persists. Furthermore, flaws in conotruncal development occur and there is a failure to achieve appropriate conotruncal maturation (inversion/rotation/looping) (2, 7, 8) (Figure 1). Different etiologies were proposed in different types of DORV by Van Praagh (9). Please refer to the Genetic associations section below.

## Morphology of DORV—sequential segmental analytic approach

**Figure 2:**
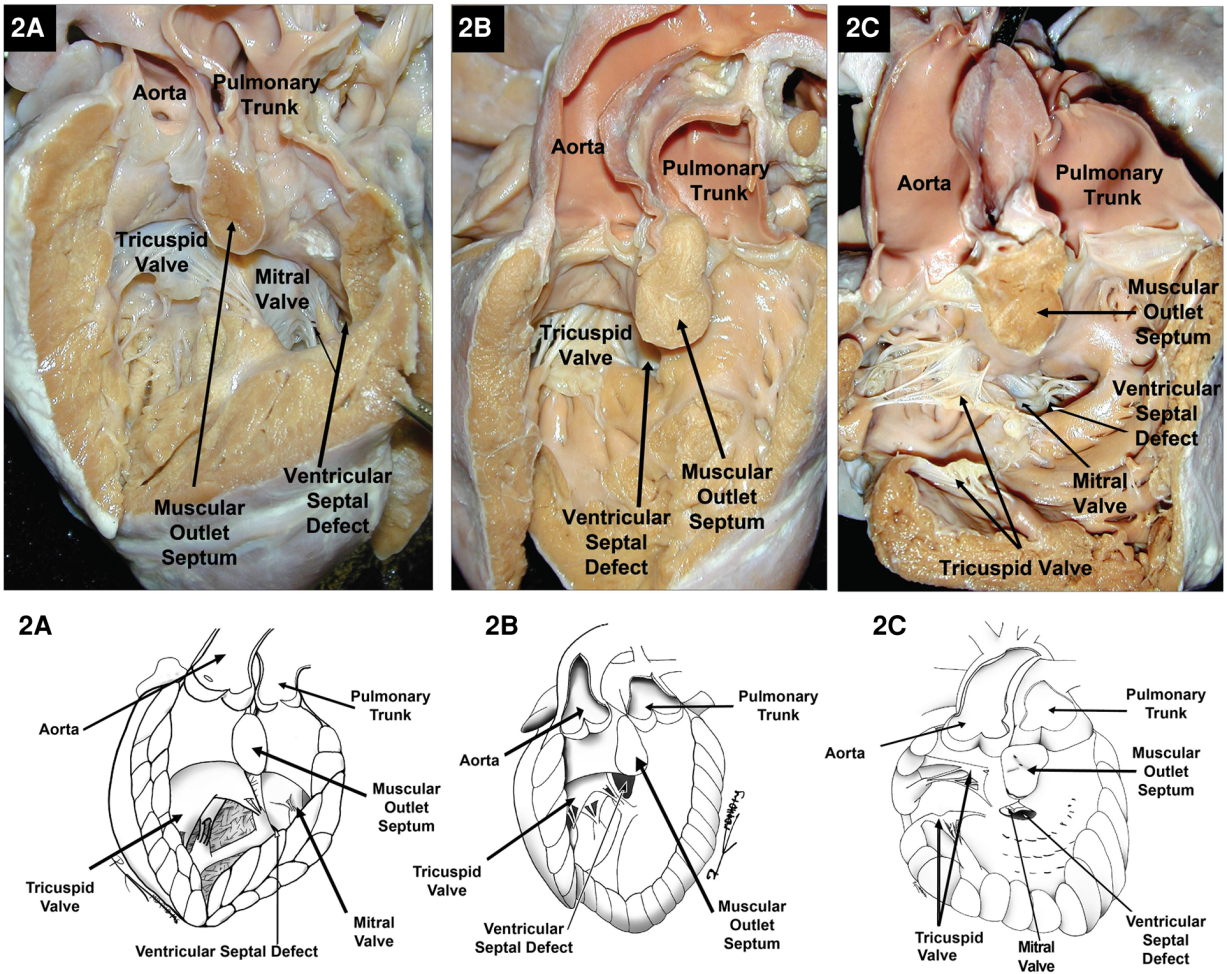
Right heart views of specimens with DORV with bilateral infundibula and the aorta to the right of the pulmonary trunk. (**A**) Subpulmonary ventricular septal defect in a Taussig–Bing-type DORV. (**B**) Subaortic ventricular septal defect with a straddling tricuspid valve. (**C**) Example of an inlet ventricular septal defect extending toward the ventricular outlets and located directly below the muscular outlet septum and both outflow tracts. Because of the location of the VSD in the inlet septum, it is arguably considered remote or uncommitted. Images courtesy of Robert H. Anderson.

**Figure 3:**
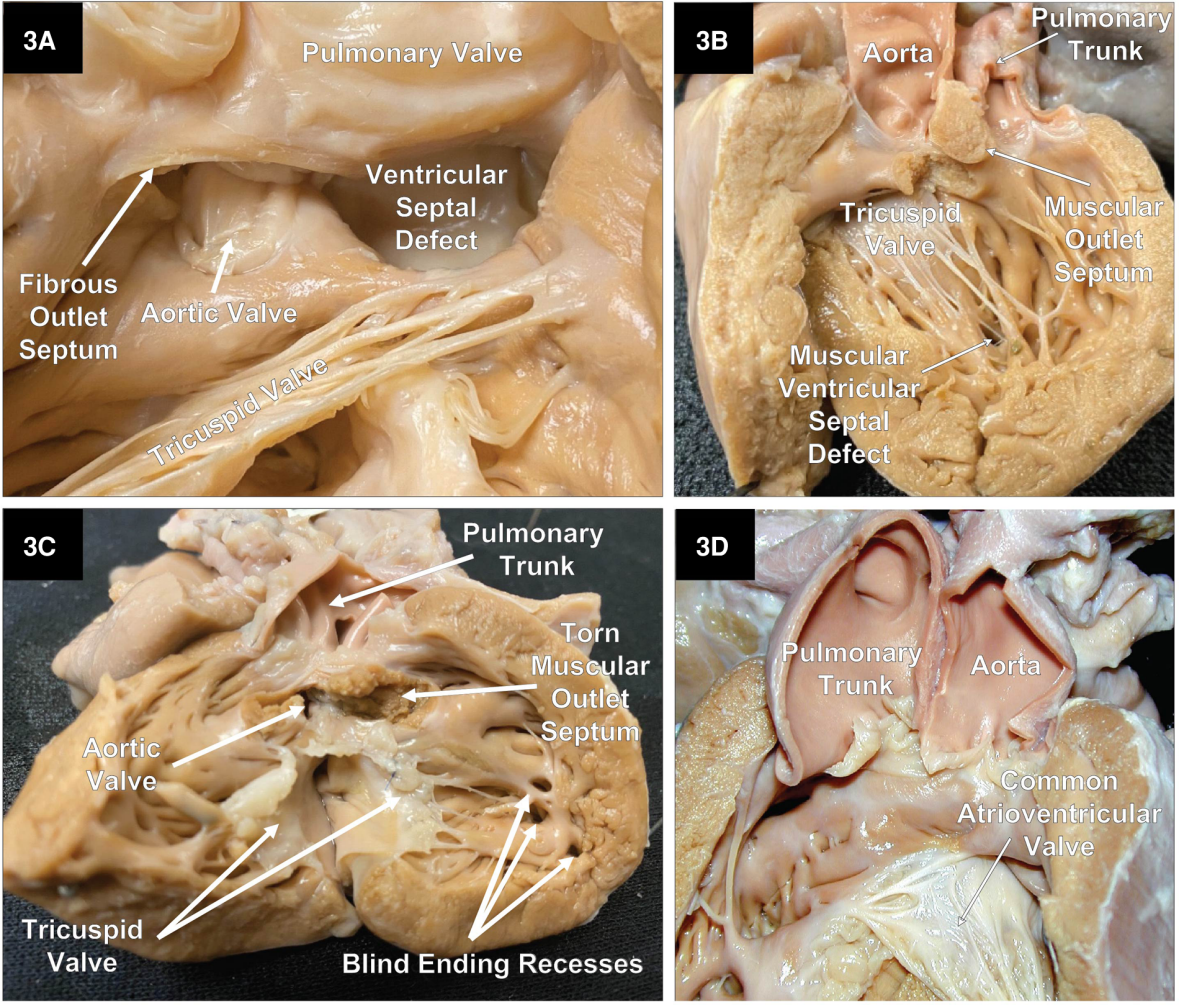
Right heart views of specimens with DORV. (**A**) A close-up of an example of a doubly committed ventricular septal defect with a small fibrous outlet septum and fibrous tissue creating continuity between the arterial valves. Part of the pulmonary valve and free wall of the pulmonary outflow tract has been reflected to the right. (**B**) A case of DORV with a small remote uncommitted apical muscular ventricular septal defect. (**C**) DORV with an intact ventricular septum and a stenotic aortic outflow tract. This heart also had mitral valve agenesis, and an atretic LV that lacks an inlet as well as an outlet. (**D**) A specimen with an AVSD with both great vessels arising from a single muscular infundibulum without a muscular or an obvious fibrous outlet septum. In addition, there is fibrous continuity between the arterial valves. Because there are two distinct arterial valves, this is not a common arterial trunk. In addition, the aorta is to the left of the pulmonary trunk.

**Figure 4:**
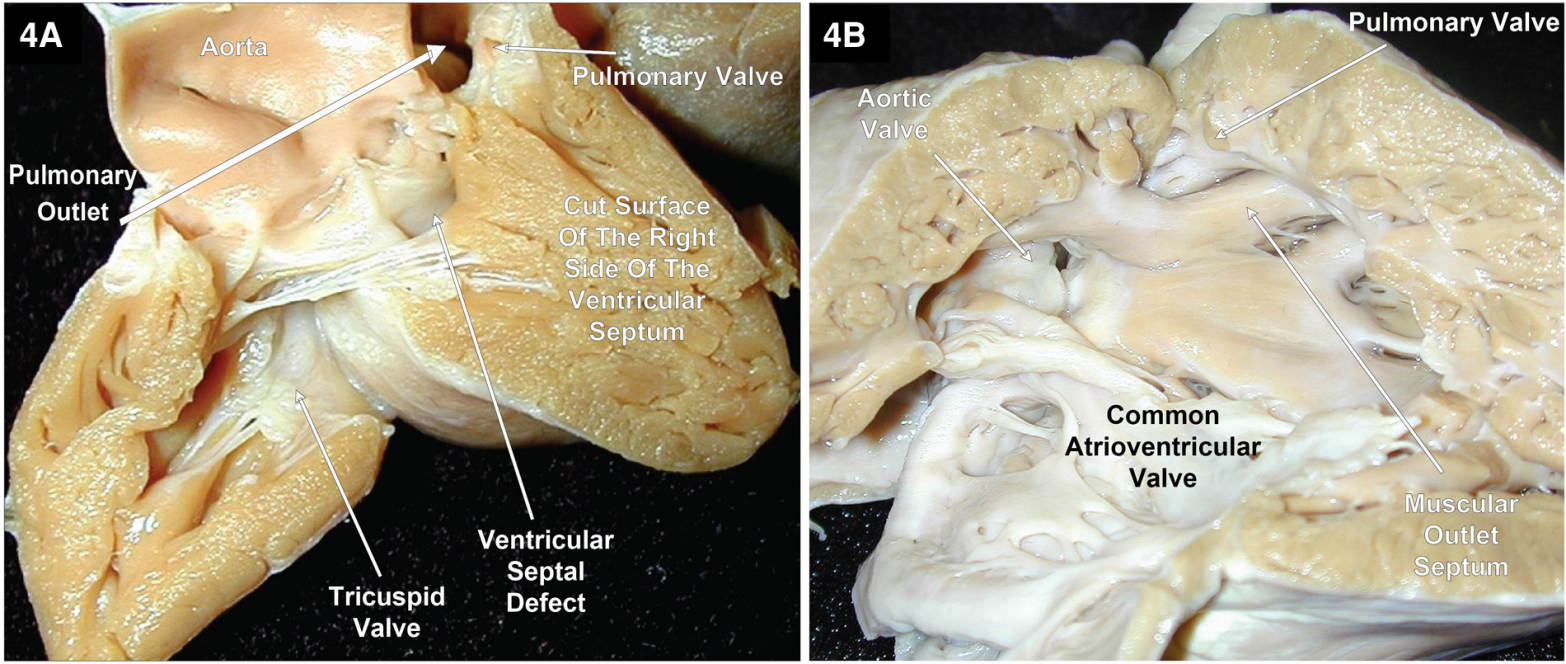
Right heart views of two specimens with TOF and DORV. (**A**) shows most of the aorta arising from the RV with the atrioventricular valves and the aortic valve in fibrous continuity through the ventricular septal defect that is located between the limbs of the septal marginal trabeculation. In addition, this patient’s twin had TOF with a slight overriding of the VSD by the aorta. (**B**) Pulmonary stenosis and the aortic valve in fibrous continuity with the common atrioventricular valve in a heart with TOF and an AVSD. Image courtesy of Robert H. Anderson.

As previously mentioned, DORV can occur when the great arteries are supported by a muscular sleeve that is commonly partitioned into by two outlets (infundibula) by a muscular or a fibrous outlet septum yielding a discontinuity between the semilunar valves and the atrioventricular valves or a common atrioventricular valve (Figures 2A–C, 3A–C). In addition, DORV can also occur with atrioventricular–arterial valvar fibrous continuity as seen in the tetralogy of Fallot (TOF) (Figures 4A,B). The latter condition is the more common type of DORV and shows that bilateral muscular outflow tracts without arterial–atrioventricular valvar continuity are not essential for the diagnosis of DORV (11).

**Figure 5:**
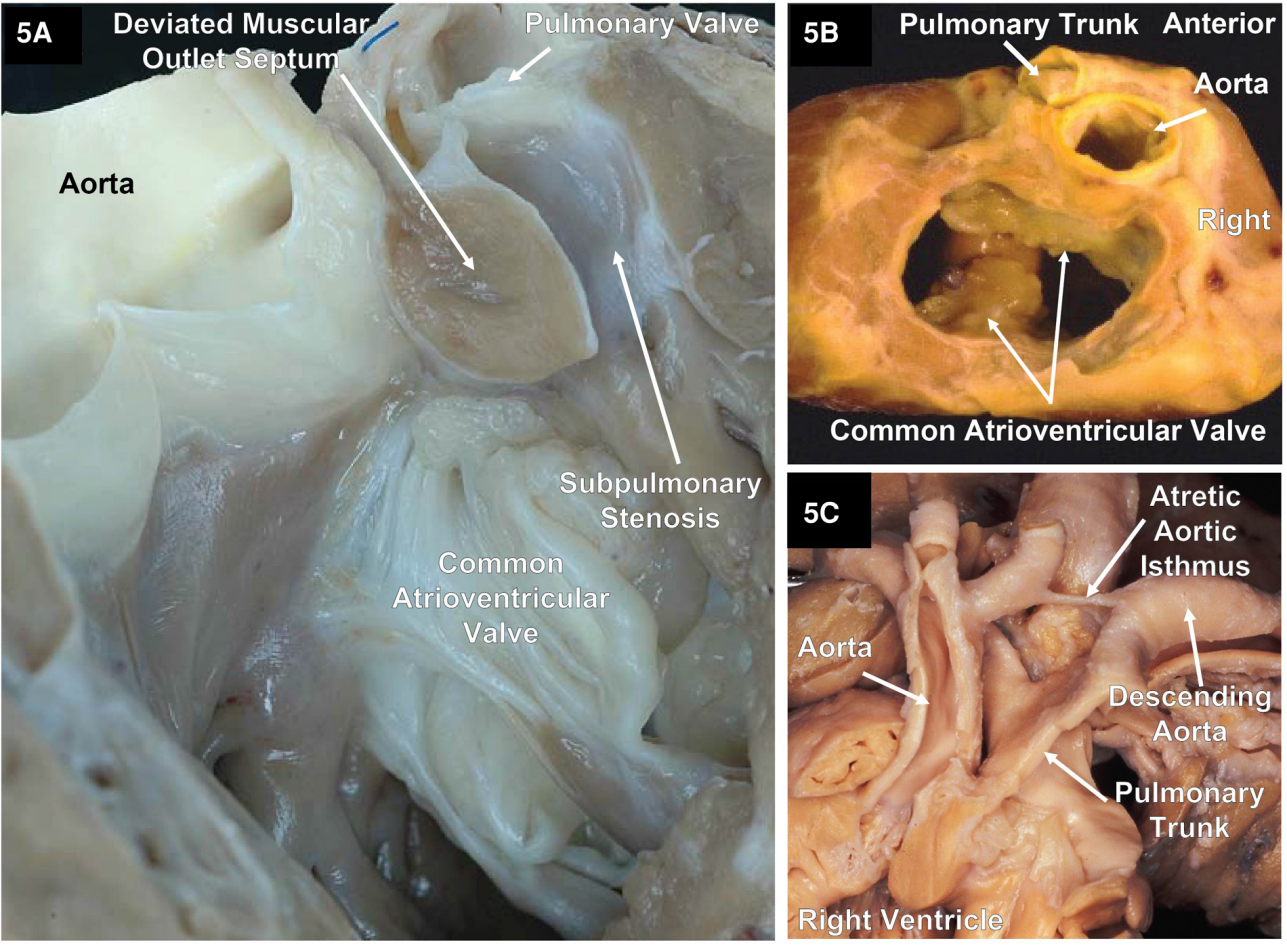
Two specimens with DORV and an atrioventricular septal defect and one specimen with DORV showing the great arteries with atresia of the aortic isthmus. (**A**) A right ventricular outlet view that illustrates the AVSD and a deviated muscular outlet septum resulting in subpulmonary stenosis. Image courtesy of Robert H. Anderson. (**B**) A base view after the atrial segment has been removed of a cardiac explant showing a common atrioventricular valve with a single orifice (complete) AVSD and DORV with normally related great arteries along with pulmonary stenosis. (**C**) The great arteries of a specimen with atresia of the aortic arch isthmus. Image courtesy of Robert H. Anderson.

In classical DORV, the outlets are partitioned by a muscular outlet septum. The displacement of this outlet septum into the aortic or pulmonary outflow tracts will create an element of subvalvar obstruction. Figure 5A shows the displacement of the muscular outlet septum into the pulmonary outflow tract creating subpulmonary stenosis. In exceedingly rare instances, both great arteries can arise from the morphologic RV supported by a single muscular outlet (sleeve) without an obvious outlet septum (Figure 3D).

The relationship between the aortic outlet and the pulmonary outlet can vary. The aortic outlet may be positioned right-or-left anterior, right-or-left posterior, directly anterior to the pulmonary outlet, or the arterial outlets may have a side-by-side configuration. Occasionally the aortic valve and the pulmonary valve may have a normal relationship (Figure 5B). In hearts with DORV in which the great arteries do not have a normal relationship to each other, it makes more sense to describe the great arteries as being malpositioned rather than TGA to avoid confusion. TGA is more accurately defined as having discordant ventriculo-arterial connections, that is the aorta arises from the morphologic RV and the PA from the morphologic LV. In contrast, in DORV, the aorta has a discordant connection while the PA has a concordant connection. Therefore, it is better to use descriptive terms to describe the relationship between the aorta and pulmonary trunk; however, some favor using the prefixes “d” (right) or “l” (left). For example, in the setting of DORV with the aorta to the right or left of the pulmonary trunk, it is more accurate to describe the condition as d-malpositioned and l-malpositioned, respectively (12).

In rare cases, where the interventricular communication is restricted or absent (Figure 3C), the egress of blood from the LV will not be through a VSD or an AVSD. In addition, the LV will show an element of hypoplasia, which can vary from mild to severe.

The location of the VSD in DORV as viewed from the morphologic RV may be subpulmonic, subaortic, doubly committed (fibrous continuity between the arterial valves), or remote and non-committed (Figures 2A–C, 3A–B). Except for the remote type of VSD, the others will be located between the anterior and posterior limbs of the septal marginal trabeculation (13).

In hearts where the VSD is subpulmonic and the pulmonary valve overrides the crest of the ventricular septum, this entity is described as a Taussig–Bing-type malformation. The classic description of the Taussig–Bing malformations corresponds to a DORV when more than 50% of the pulmonary valve overrides the morphological RV, the semilunar valves are side-by-side, and there is a bilateral conus with approximately equally well-developed conal free walls and a subpulmonic VSD (4). If the majority, more than 50%, of the pulmonary valve sits above the morphologic LV, this would be considered a TGA because the pulmonary trunk has a discordant ventriculo-arterial connection. Conversely, if most of the pulmonary trunk overrides the morphologic RV, this type of heart would be a Taussig–Bing type DORV, and the ventriculo-arterial pulmonary connection is considered to be concordant (Figure 2A). The presence or absence of pulmonary-mitral continuity is another important feature in differentiating TGA with a perimembranous VSD and Taussig–Bing malformation. In TGA with a perimembranous VSD and overriding PA, the pulmonary–mitral continuity is present, whereas in the Taussig–Bing malformation, the pulmonary–mitral continuity is absent.

As Dr. Robert H. Anderson has alluded, the definition of a DORV cannot rely on a single phenotypic feature, as is the case of the presence of bilateral conus in the outlets (14). DORV also occurs when one great artery arises from a muscular infundibulum and the other great artery arises entirely or mostly from the RV, and its valve is in fibrous continuity with the atrioventricular valves or a common atrioventricular valve. An example of this would be TOF with DORV with a VSD or an AVSD (Figures 4A,B).

If both great arteries are connected totally or mostly to the right ventricular component of a heart, no matter what the condition of the great arteries, even if one is atretic, and the entire egress of blood from the RV is through only one great artery (single outlet), morphologically, DORV is present. One example would be TOF with DORV and pulmonary atresia.

DORV with a hypoplastic LV has been classified as a variant of hypoplastic left heart syndrome (15) if there is also obstruction of the left atrioventricular connection and the systemic outflow tract, but this is not a universally accepted concept.

A wide variety of cardiac malformations of the heart may accompany DORV including coarctation of the aorta (CoA), severe mitral valve malformations like mitral atresia, laterality defects such as the heterotaxy syndromes of isomerism of the atrial appendages (16, 17), aortic and pulmonary stenosis (18), interrupted aortic arch (IAA) and aortic arch obstruction (19, 20) like hypoplasia or atresia of part of the aortic arch (Figure 5C), bicuspid aortic valve (21, 22), and double inlet–double outlet right ventricle (23) and straddling tricuspid valve (TV) (Figure 2B).

## Classification/pathophysiology

The most frequently used classification of DORV is based on the location of the VSD and the position of great arteries (5), the relationship of great arteries to each other, and the presence or absence of outflow tract obstruction (Table 1). Given that the traditional classification did not correlate with the perioperative mortality or complexity of the required surgical repairs, DORV can be considered through a functional classification based on physiological characteristics that help determine the surgical approach they will be required.

**Table 1:** DORV categories based on VSD location and the relationship with great arteries (5).

Subaortic VSD	
Subpulmonary VSD	
Doubly committed VSD	
Non-committed VSD/remote VSD	
DORV categories based on great vessel relationship	
Right anterior aorta	Left anterior aorta
Right posterior aorta	Left posterior aorta
Right lateral/side-by-side aorta	
DORV categories by Van Praagh (9)	
Type I DORV as an isolated conotruncal anomaly	
Type II DORV with conotruncal anomalies and associated malformations of the atrioventricular valve(s) and ventricles	
Type III DORV associated with heterotaxy	
Functional categories by congenital heart surgery nomenclature and database project (3)	
DORV, VSD type (DORV-VSD)	
DORV with subaortic or doubly committed VSD and pulmonary stenosis, Fallot type (DORV-Fallot)	
DORV, with subpulmonary VSD, transposition type (DORV-TGA)	
DORV, non-committed VSD (nc-VSD)	

A subpulmonary VSD *in utero* could lead to less flow along the LV and left ventricular outflow (LVOT) leading to subaortic/aortic stenosis, CoA and IAA. A subaortic VSD may mean that pulmonary stenosis and small branch pulmonary arteries are likely to develop. Not surprisingly, outflow tract obstruction can be seen, either left ventricular outflow tract obstruction (LVOTO) or right ventricular outflow tract obstruction (RVOTO). Some other common associations include AV valve anomaly: straddling vs. atresia, as well as common AV valves can be found with DORV. Coronary abnormalities may be similar to those seen in TOF and TGA, as well as the associated abnormalities with heterotaxy.

## Genetic associations

DORV is reported to occur in approximately three to nine in 100,000 live births. Like many complex congenital heart defects, DORV may occur as an isolated defect as well as with other cardiac and extracardiac anomalies (9, 24–28). During cardiac development, the outflow tract connects exclusively to the primitive RV; after the extensive and complicated process of remodeling, the outflow tract will divide into two separate great arteries: the pulmonary artery and the aorta. Further remodeling then progresses to establish the connection between the LV and the aorta (29). The pathological mechanisms by which specific genetic anomalies or environmental exposures result in the development of DORV from normal structural cardiac anatomy are unknown. A variety of chromosomal and syndromic abnormalities are associated with individuals with DORV:

1. Chromosomal abnormalities: More than 40% of patients with DORV have associated chromosomal abnormalities; most of those patients also have extracardiac defects (25).
  a. Aneuploidies: Observation from epidemiologic data (30) and necropsy studies (31) support an increased risk of DORV in trisomy 13 and 18 with the association of hypoplasia of left heart structures. Although previous studies did not observe a heightened risk in trisomy 21 (30, 31), Obler et al*.* noted that trisomy 21 is found in 4% of DORV patients (25). DORV with left ventricular hypoplasia has also been reported in a patient with a 47, XYY genotype (32).
  b. Copy number variations: Cytogenetic analyses have identified that discrete chromosomal regions are associated with the pathogenesis of various congenial heart defects (33, 34). DORV has been reported in the following copy number variants: Chromosome 8 abnormalities have been identified in 10% of DORV patients. 22q11.2 deletion syndrome presents in 7% of DORV individuals (25). 5p15.2 deletion (Cri-du-chat syndrome) (35) and Jacobsen syndrome (11q terminal deletion) (36) have been reported related to DORV (37).
2. Single gene defects
  a. Transcription factors that are crucial during the cardiac development.
  b. There are three cell lineages in the cardiac outflow development: (1) distal mesenchymal cells originating from the cardiac neural crest cells; (2) the conal cushions in the proximal outflow tract mesenchymal cells, which originate from the endocardium via endocardial–mesenchymal transition (EndoMT); and (3) precursors from the first and second heart fields. The first and second heart field cells are the mesodermal cells that differentiate into cardiomyocytes from the initial heart tube.
  Mutations in human and mouse models with DORV have identified that multiple genes relate to the intriguing process of these cell lineages to develop a mature and separated outflow tract (38) including *NKX2.5*, *FOG2*, *TBX5*, and *TBX1* (25, 37). Mutations in *TBX5* have been identified in patients without the 22q11.2 deletion syndrome (39).
  c. It has been hypothesized that Van Praagh type I DORV might reflect the defects of cardiac neural crest cell migration from the hindbrain to the cardiac outflow tract, or the abnormalities in the cardiac neural crest cells and second heart field cells (25). Abnormalities in genes that regulate the *TGF*-β family (40), *Wnt* pathway (41), *BMP* pathway (42) and related to cardiac neural crest development and migration (25) [*Sox*-4 (40), Retinoid pathway (43), *ECE*-1 (44), *ECE*-2 (44), *Pax3* (45), maternal diabetics (25, 30, 46), *LRP1* (47)] have been observed to be related to DORV. To support this hypothesis, our group previously reported the evidence of the perturbance of cardiac neural crest lineage that resulted in DORV in a mouse model of *Lrp1* mutation (47).
  d. Van Praagh type II DORV may be related to the consequence of developmental abnormalities of the endocardial cushions such as mutations in *GATA4* (48).
  e. The left–right patterning during the cardiac development is established during early embryogenesis by the multiple interplays of different signaling pathways and by the action of the nodal cilia (49). Since Van Praagh type III DORV is associated with heterotaxy, it is reasonable to predict that genes or teratogens that altered left–right determination or ciliopathies (50) are candidates for type III DORV such as *Pitx2* (51), *CFC1* (52), *Cx43* (53, 54), *Vangl2* (55), *lefty1* (56), and *inversin* (50).
3. Epigenetic regulators: Two general issues have been observed with the advances in genomic technologies to understand the molecular genetic bases underlying the congenital heart defects: a single genetic abnormality can be associated with different types of congenital heart defects and similar cardiac lesions have been associated with different chromosomal regions, genes, or signaling pathways (33). In addition to the biology of the genome, the contribution of epigenetic mechanisms to cardiac development is acknowledged (57). For example, mutations in the *CHD7* genes result in CHARGE association with the perturbation of the H3K4 methylation (58). Congenital heart defects occur in 75%–80% of the patients with CHARGE association including DORV (59). In addition, an enrichment of *de novo* mutations in histone-modifying enzymes required for H3K27 methylation were noted in 362 severe CHD patients, which included DORV patients (60).

## Investigation/diagnostic studies

### Electrocardiogram

Due to the heterogeneous nature of DORV, there is no pathognomonic electrocardiogram (EKG) pattern. In fact, the EKG may often be normal or demonstrate only nonspecific findings. However, in the most common subtype, the TOF subtype or sub-aortic VSD with PS, the EKG may show right axis deviation with right ventricular hypertrophy (denoted by rR’, rsR’, or qR pattern in V1) similar to classic TOF (61). Similarly, DORV in the setting of AVSD may show superior QRS axis in the frontal plane with prolongation of the PR interval due to posterior displacement of the conduction system as seen in classic AVSD. The presence of heterotaxy with atrial isomerism may result in rare findings of twin AV nodes in right atrial isomerism denoted by two distinct QRS morphologies. Left atrial isomerism may reveal a superior *P* wave axis or AV conduction deficits. In general, atrial isomerism is a rare finding; however, DORV is not an uncommonly described ventriculo-arterial arrangement in this population. This was originally described as the Van Praagh class III with right/left imbalance and heterotaxy (9). There is also a higher incidence in Asian populations related to higher incidence of right atrial isomerism (62). Congenital AV block is a rare finding in common subtypes of DORV but may have a higher incidence in the setting of additional cardiac malformations including AVSD (61) and left atrial isomerism (63).

### Echocardiogram

#### Fetal echocardiogram

Gottschalk et al. reported a single center tertiary institution study over an 8-year consecutive period where all of their DORV cases were prenatally diagnosed (26). There were 46 cases in all and 96% of them were correctly diagnosed. There were 17 pregnancy terminations, two intrauterine deaths, and nine post-natal deaths. There were 21 children who underwent repair, including eight patients who had biventricular repairs and 13 patients who had univentricular palliation. Interestingly, all children received their prenatally predicted type of repair. The authors’ conclusion was that though it was rare, DORV can be diagnosed prenatally with impressive accuracy for surgical planning and parental education. As such, fetal echocardiography plays a crucial role in prenatal diagnosis. By answering critical questions about the location of VSD, the relationships of the great arteries, the sizes of respective ventricles, outflow tract anatomies, and coronary anatomy, it can predict if the condition can be treated with biventricular repair or if it needs univentricular palliation.

#### Transthoracic echocardiography

Once the baby is born, transthoracic echocardiography remains the diagnostic modality of choice. It provides an accurate delineation of the intracardiac anatomy and allows for the assessment of the associated hemodynamics. The location of the VSD relative to the semilunar valves is key to surgical planning and management. The echocardiogram can also reveal specifics such as the degree to which the aorta is committed to, and overriding the VSD, and ensure that the atrioventricular valve chordal attachments do not straddle the VSD.

**Figure 6:**
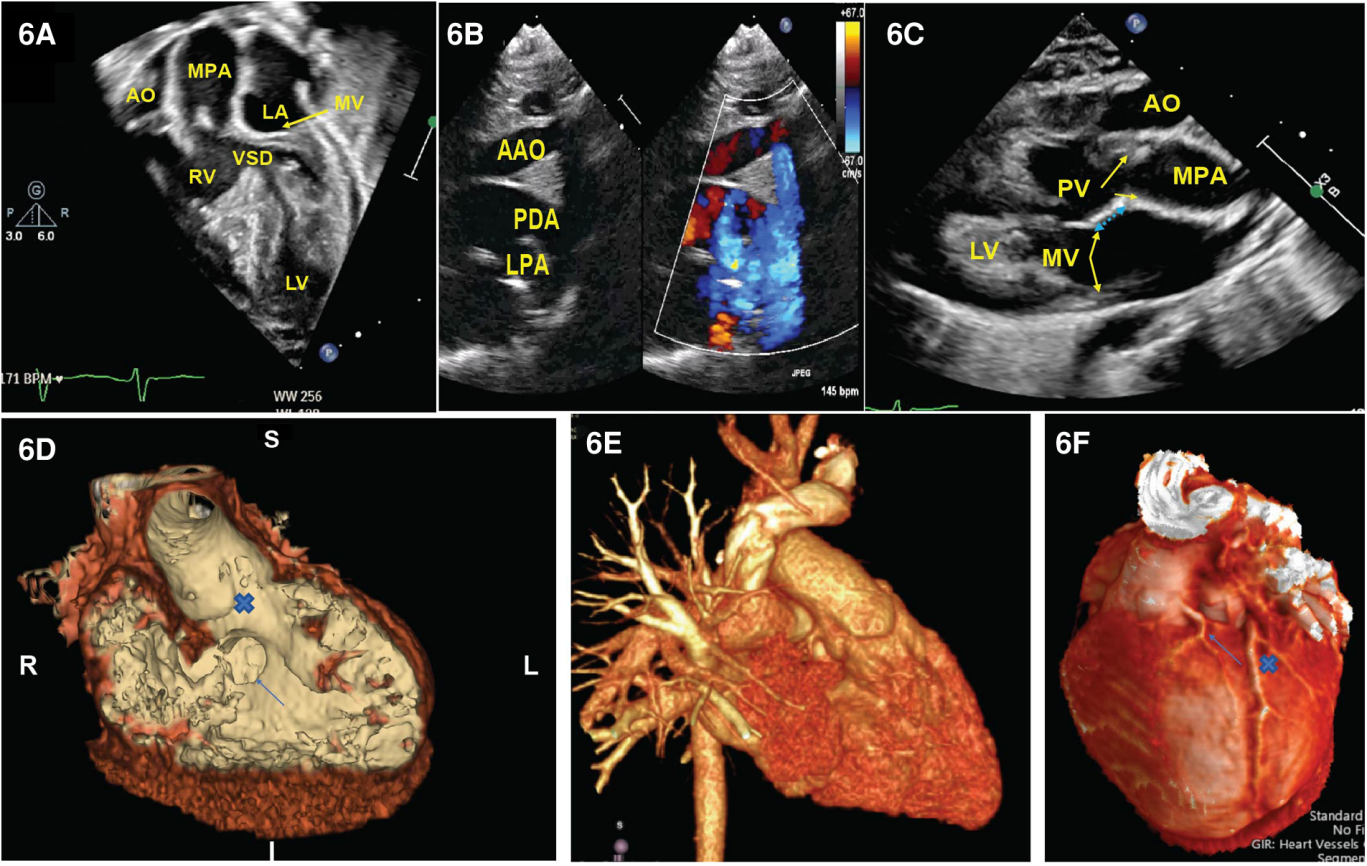
Representative images of DORV. (**A**) Four-chamber view demonstrated aorta is right to the PA and a VSD is beneath the pulmonary valve. (**B**) Supra-sternal view demonstrated arch hypoplasia with a large patent ductus arteriosus. In (**C**), we appreciate a parasternal long-axis view where the aorta is anterior to the PA with loss of the aorto-mitral valve continuity (blue arrow). (**D**) CT angiogram endoluminal view. A view of the VSD from the right ventricular side (arrow) and the VSD to aortic pathway X. (**E**) 3D reconstruction of the heart showing the anterior origin of the aorta and the origin of the left coronary artery. (**F**) Dual LAD arising from the leftward-facing sinus (arrow) that courses down the interventricular groove parallel with the larger retropulmonary LAD (x). LAD: left anterior descending artery.

The echocardiographic images can highlight the size and location of the VSD and the pathway for the surgeon to connect one of the arterial trunks with the left ventricle. Furthermore, it can help identify the aortic origins of the coronary arteries and any additional abnormalities, such as intra-atrial communications, aortic arch abnormalities, and the presence of additional VSD. Echocardiography can also clarify the anatomy of the subaortic and subpulmonary conus, which is important for planning the surgical connection. Figure 6 demonstrates a series of echocardiographic pictures in a patient with DORV/Taussig–Bing anomaly.

### Computed tomography

Computed tomography (CT) stands as a powerful diagnostic instrument for both preoperative and postoperative evaluation of DORV. It offers certain advantages over echocardiography and magnetic resonance imaging (MRI), such as the absence of limitations related to a small acoustic window or the need for anesthesia (64). Furthermore, CT imaging can also be applied to patients with metallic implants. With the current generation of CT scanners providing high spatial and temporal resolution, broad detectors, high-pitch scanning mode, dose-reduction algorithms, and advanced three-dimensional post-processing tools, CT serves as a safer and high-quality alternative to diagnostic cardiac catheterization or MRI. Consequently, CT imaging has seen increasing use in the diagnosis and management of virtually every form of congenital heart defect, but it is particularly useful in DORV. CT can also identify the coronary artery origins. Three-dimensional displays and printing of computed tomography reconstructions can assist in the detailed analysis of the ventricles’ connection and the pathway from each ventricle to an arterial trunk. This kind of detailed imaging has proven to aid in determining the optimal surgical treatment for complex cases (Figure 6).

### Angiogram/cardiac catheterization/CT

The purpose of additional studies in addition to an echocardiogram would be to clarify the details of the anatomy such as branch pulmonary arteries, arch anatomies, and coronaries; it may also assist in clarifying the location of the VSD.

### Pre-operative management

Pre-operative management is highly dependent on the patient’s hemodynamics. For instance, patients with subaortic VSD or subpulmonary VSD can have pulmonary over-circulation requiring aggressive diuresis. At times, this may lead to the need for respiratory support and intubation. If there is too little pulmonary blood flow, prostaglandin E1 infusion and/or atrial septostomy should be considered. A subset of patients will have adequate pulmonary blood flow initially and they can be sent home to grow and await later surgical intervention. These patients may develop increasing cyanosis over time and may even develop hyper-cyanotic spells like patients with TOF. These patients will need their patent ductus arteriosus (PDA) to be kept patent, either with prostaglandin E1 administration or PDA stent placement; or sometimes they may need the placement of a central shunt. Consideration should be given to the urgent need for atrial septostomy in a patient who is desaturated and has evidence of a poor cardiac output state. If the patient has significant aortic stenosis or coarctation of the aorta (9, 64), then they may present with cardiogenic shock, which may require inotropy, cardiac catheter-based intervention, and even veno-arterial extracorporeal membrane oxygenation support.

### Surgical management

DORV represents an extremely heterogeneous group that requires a variety of surgical approaches, considerations, and techniques for proper repair. The spectrum of surgical techniques spans from biventricular repairs, which include intraventricular rerouting patch, Rastelli-type repair (with RV to PA conduit), root translocations and the arterial switch operation (ASO), or univentricular staged palliation procedures (Table 2).

**Table 2:** Criteria for single or biventricular repair of DORV.

Univentricular	Biventricular
Small/atretic atrioventricular valve, straddling atrioventricular valve	Normal/nearly normal atrioventricular valve
Remote VSD	VSD that can be incorporated into a baffle
Hypoplastic ventricle	Normal ventricular size
Abnormal coronary anatomy	Conductive coronary anatomy

Some important anatomical features to considered when planning the surgical repair of DORV include the following:

1. The relationship of the atrioventricular conduction axis to the margin of the VSD
2. The spatial relationship of the VSD to the septal leaflet of the tricuspid valve, and atrioventricular valve abnormalities
3. The size of VSD
4. The presence and extent of the muscular infundibulum
5. The orientation of the conal septum in relation to the VSD margin
6. The volume/size of ventricles
7. Any associated abnormalities (such as coronary artery anomalies)

The combination of these anatomical features determines if a patient can undergo a biventricular repair or a univentricular palliation. Some of the features pointing to either pathway are listed in Table 2.

In addition, some patients may require palliative procedures before a definitive surgical repair, especially in premature neonates; a syndromic infant with heterotaxy; a baby who is small for gestational age/with intrauterine growth restriction (IUGR); and/or infants with significant extracardiac abnormalities such as renal or intracranial issues. Such palliative procedures include PA band placement for babies with pulmonary over-circulation, PDA stent, or operative central shunt creation for those with inadequate pulmonary blood flow. A consideration in the modern era is the utilization of the hybrid procedure to optimize the Qp/Qs.

## Biventricular approach

### DORV-VSD type

Surgical management of these patients commonly involves the creation of an intraventricular baffle. It is recommended to undergo repair within the first 6 months of life to limit the long-term effects of pulmonary over-circulation. When a delay is foreseen, pulmonary band placement can be pursued before a complete repair occurs at a later time.

**Figure 7:**
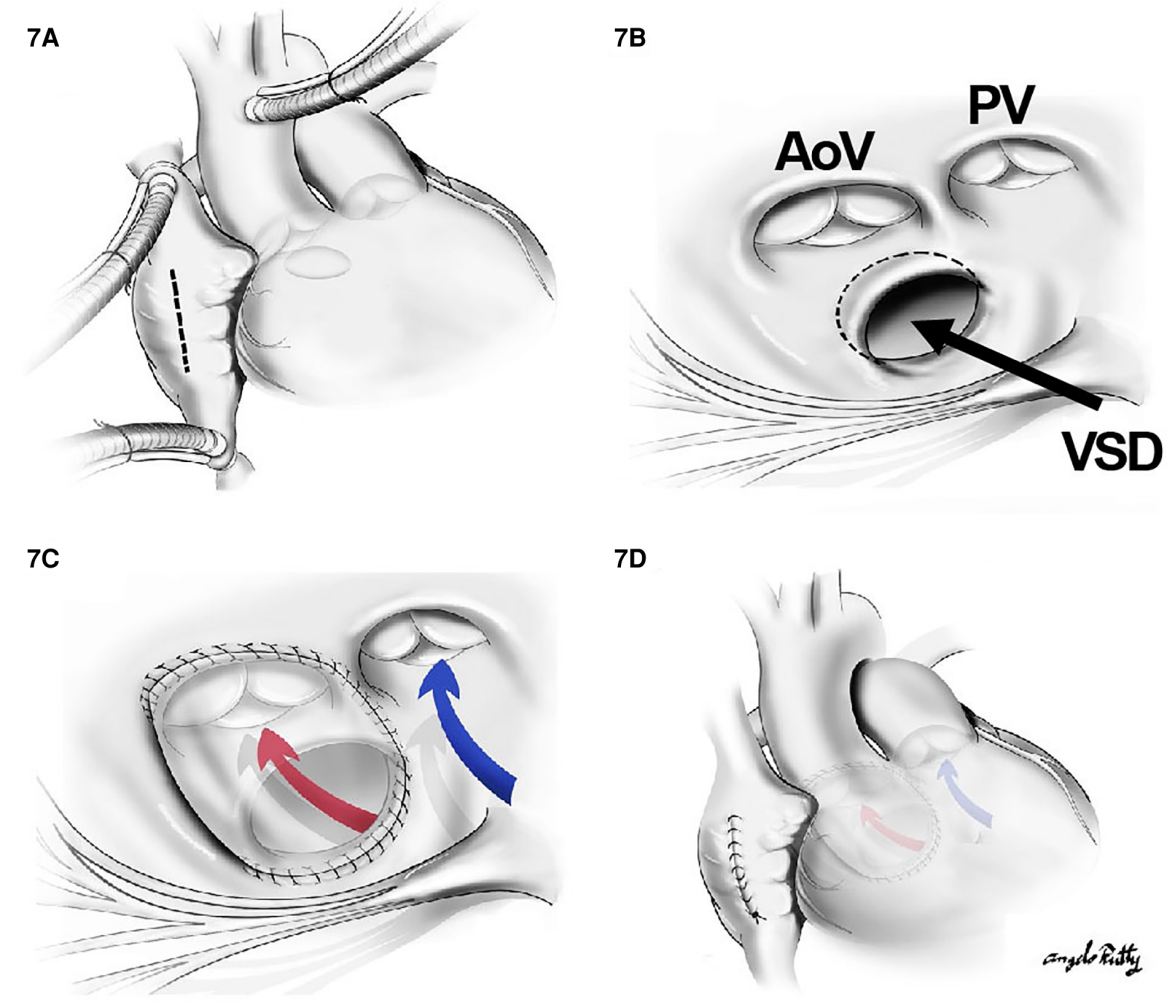
The repair of a subaortic VSD: (**A**) a cut through the right atrium, (**B**) surgical view of the subaortic VSD in the relationship with aortic valve (AoV) and PV as well as the papillary muscle of the tricuspid valve. In (**C**) and (**D**), a straight tunnel is created by using a VSD patch through LV to the aorta (red arrow). The blue arrow indicates the flow from RV to PA.

**Figure 8:**
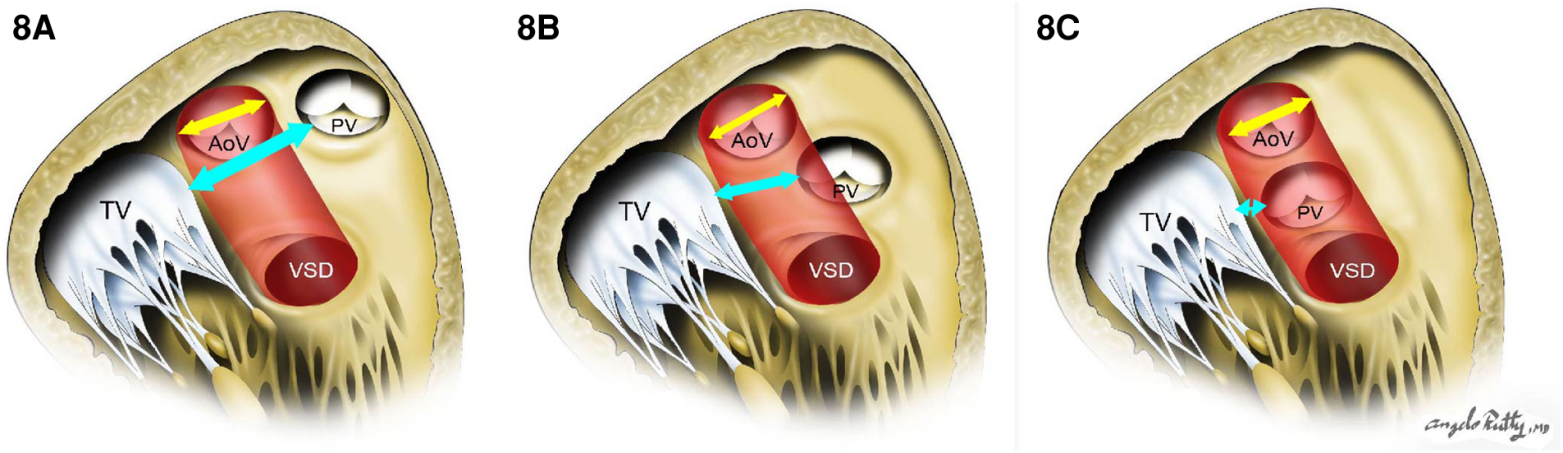
The risk of pulmonary outflow tract obstruction with the intraventricular baffle when distance is too short. (**A**) and (**B**) show low risk of POTO: the distance between tricuspid valve and pulmonary valve (cyan arrow) is more than the aortic opening diameter (yellow arrow). In (**C**), there is a high risk of POTO with the distance between tricuspid valve and pulmonary valve (cyan arrow) being is less than the aortic opening diameter (yellow arrow).

When the VSD is perimembranous or subaortic, a relatively straight intraventricular tunnel can be created between the VSD and the aortic valve (Figure 7). In this scenario, the relationship between the tricuspid and semilunar valve plays an important role predicting the risk of pulmonary outflow tract obstruction (POTO) (65, 66). This relationship is described by four grades: 0: fibrous continuity, 1: slight discontinuity, 2: discontinuity but distance < aortic orifice diameter, and 3: Discontinuity but distance > aortic orifice diameter. Since the tunnel will pass inferior and to the right of the pulmonary valve, it is accepted that an intraventricular repair is suitable without POTO when the distance between the tricuspid and pulmonary valve is graded as a 3 or the tricuspid to pulmonary valve distance is greater than the tricuspid to aortic distance (Figure 8). Unobstructed intraventricular baffle is usually achievable beyond the age of 3 months and the repair can be performed through the right atrium. If the VSD is restrictive, which is defined as less than the diameter of aortic annulus, enlargement of the communication is advisable to avoid the development of left ventricular outflow tract obstruction. This is carried out by resecting the antero-superior portion of the ventriculo-infundibular fold and the conal septum.

When the VSD is doubly committed, the creation of an intraventricular tunnel will likely need to be performed through a right ventriculotomy or through the pulmonary artery (67, 68). This is because the intraventricular channel is near the infundibular septum, underneath the semilunar valves, with absence or near absence of the conal septum. Resection of this remnant tissue should be done. We must keep in mind that the baffle is anchored to the fibrous tissue between the pulmonary and aortic annulus and thus there is a risk for semilunar valve distortion. Patch enlargement for closure of the ventriculotomy is necessary to prevent POTO.

### DORV-Fallot type

**Figure 9:**
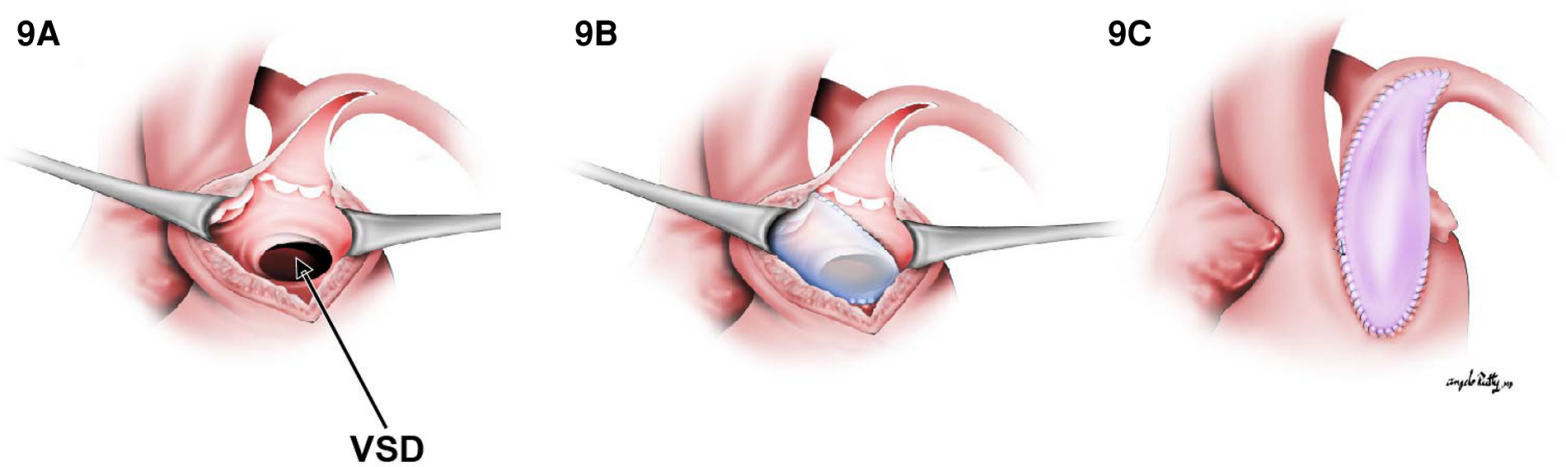
The transannular patch in DORV/TOF type. (**A**) Surgical view through right ventriculotomy. In (**B**), a straight tunnel is created by using a VSD patch through LV to the aorta. (**C**) A right ventricular outflow tract reconstruction with a transannular patch.

For this subset, the same principles for creating an unobstructed intraventricular tunnel from the LV to the aorta are utilized in addition to the strategies used to relieve general pulmonary outflow tract obstruction. Similar to TOF, the associated POTO can be sub-valvar, valvar, or supra-valvar. In scenarios where the obstruction is at the sub-valvar level, a right ventricular infundibular patch enlargement should be considered, whereas if the right ventricular outflow tract (RVOT) obstruction is instead at the valvar level, a Rastelli-type repair with an RV-to-PA conduit is indicated. Assessment of the annulus size on echocardiography as well as visually through the pulmonary trunk to determine if the *z* score is <−2 will help determine whether a transannular incision is warranted. In cases where a valve-sparing repair cannot be performed, a transannular incision is extended from the prior ventriculotomy. Transannular repair can be performed using a polytetrafluoroethylene (PTFE) monocusp valve, a valved conduit (Rastelli-type), or a valveless reconstruction (Figure 9).

Any additional obstruction produced by septomarginal and septoparietal trabeculations should be resected out. Invariably, this type of DORV is better approached directly through the ventricle due to enhanced visualization. Importantly, the patient’s acuity is determined by the degree of POTO. In this sense, symptomatic neonates might require prompt palliation with a systemic to PA shunt or a PDA stent before complete repair, whereas asymptomatic patients could undergo an elective repair at 3–6 months of age.

### DORV non-committed VSD (remote VSD)

**Figure 10:**
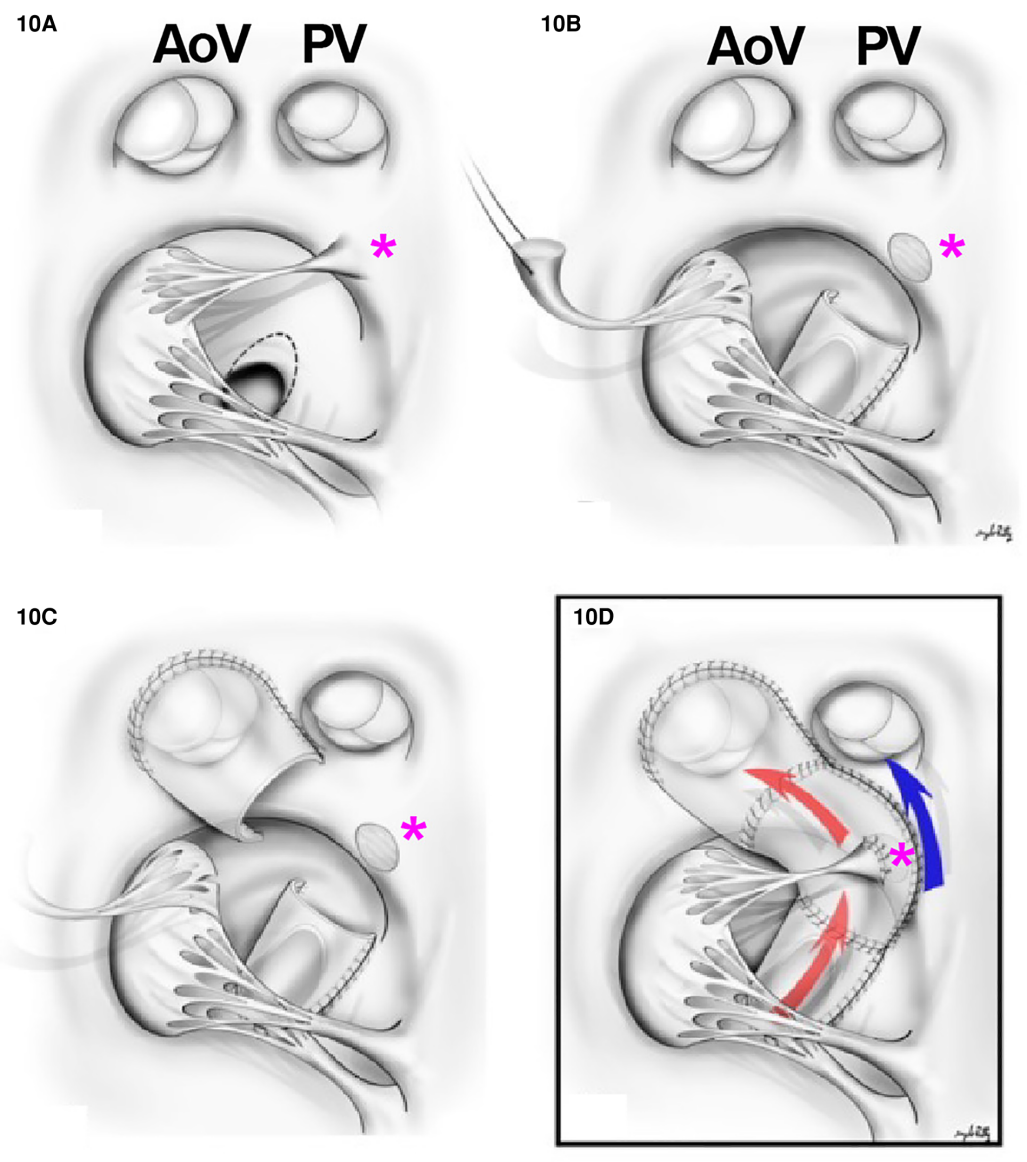
Multiple patch repairs in remote VSD. (**A**) Surgical view of the VSD with the relationship between aortic valve (AoV), PV, and the papillary muscles of the tricuspid valve (pink asterisk). (**B**) A cut through the superior papillary muscle of the tricuspid valve and a tunnel between LV to aortic valve is created in the VSD portion. (**C**) A tunnel is created to the aortic valve. (**D**) An LV to aortic valve tunnel is created (red arrow) and the superior papillary muscle is reimplanted to the patch (pink asterisk). The flow from RV to pulmonary valve is indicated in blue. *Pink asterisk, superior papillary muscle.

Often these patients will require a palliative procedure initially, either with one of three options: a systemic shunt, a PDA stent if POTO is present, or a pulmonary band placement to prevent pulmonary over-circulation. In patients with DORV non-committed VSD (nc-VSD) without POTO, biventricular repair could be accomplished with the creation of an intraventricular tunnel (VSD to aorta) if the tricuspid valve apparatus was anatomically amenable to rerouting. The main principles previously discussed regarding intracardiac tunnel are also applicable here. The main difference is that multiple patches may be needed to construct the intracardiac baffle (69) (Figure 10). Nevertheless, the route of the tunnel must be as short as possible to decrease long non-contractile areas within the left ventricular outflow tract and to avoid impinging upon the tricuspid valve apparatus. Alternatively, in patients with DORV remote VSD without valvar POTO, a VSD to PA baffle creation and ASO is preferred. The creation of a baffle between the VSD and the PA in conjunction with an arterial switch operation provides a shorter tunnel and is not impacted by the tricuspid valve (70). The VSD is always enlarged by resecting out the superior and the anterior rim. If the tunnel obstructs the right ventricular outflow tract, an infundibular patch should be placed.

However, in the presence of pulmonary obstruction, or when the intraventricular tunneling is not desirable, double root translocation can be considered for DORV remote VSD.

### DORV-TGA type

**Figure 11:**
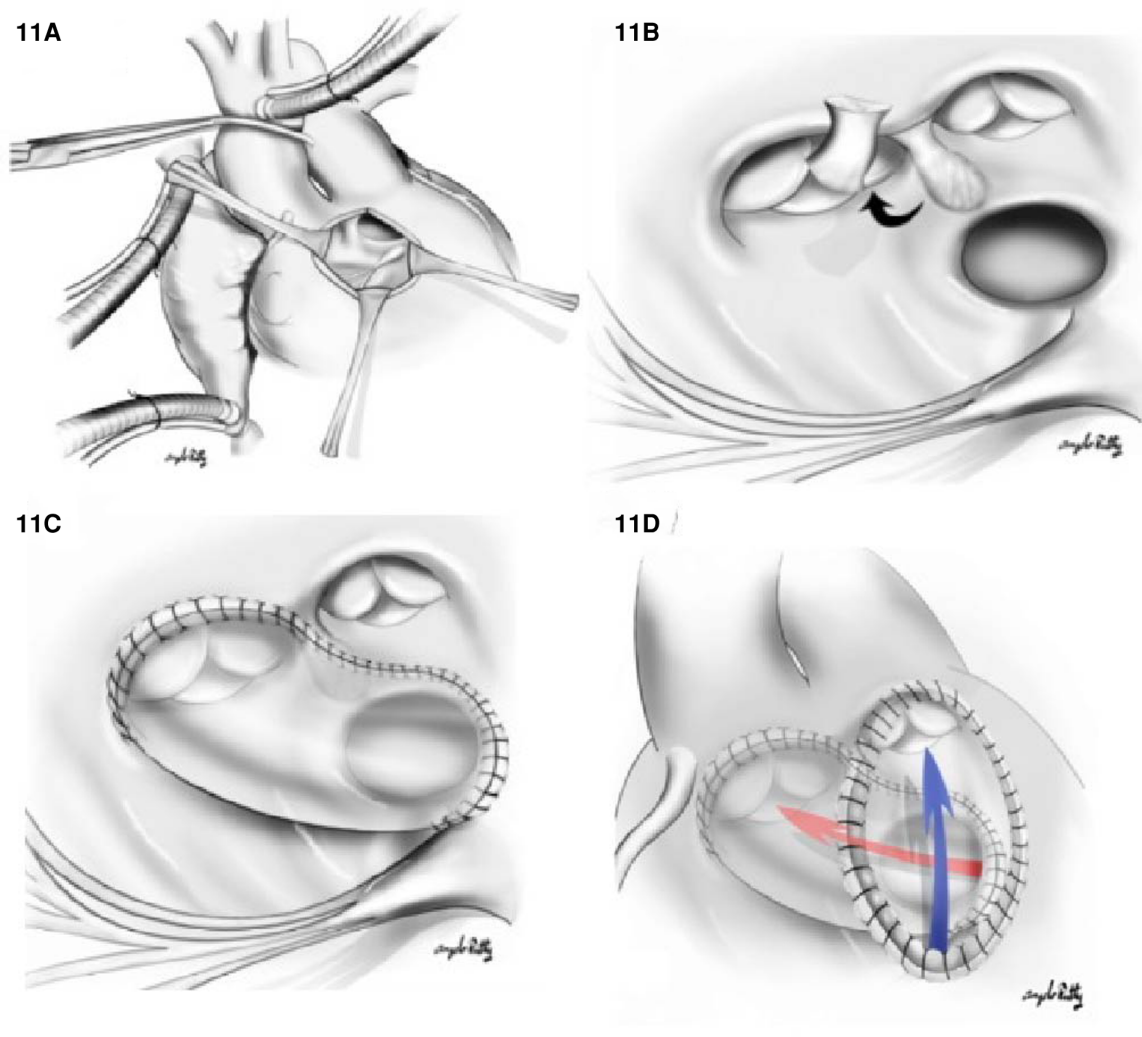
Kawashima repair of DORV with (**A**) right ventriculostomy. (**B**) A surgical view of the relationship between VSD and the aortic valve (AoV), PV, and the papillary muscle of tricuspid valve. The conal septum is resected (black arrow) to enlarge the VSD. In (**C**), a tunnel is created from LV through VSD to aortic valve. In (**D**), we appreciate a ventriculotomy patch used to increase the RVOT volume; the blue arrow indicates the direction of flow from the RV to PA.

**Figure 12:**
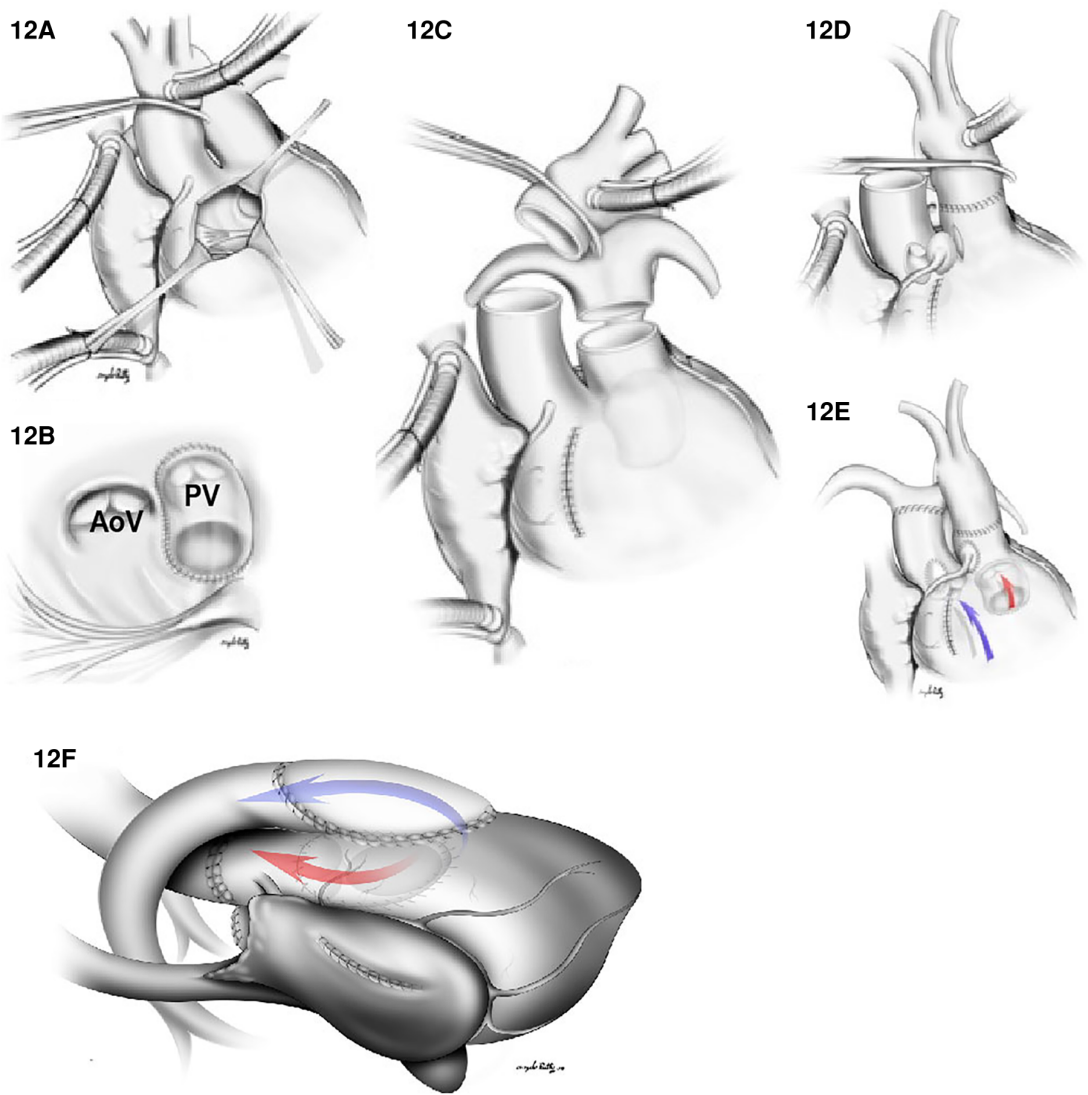
(**A–E**) Arterial switch and VSD closure for the DORV/TGA type repair. (**A**) Right ventriculotomy. (**B**) A VSD patch is placed between VSD and the native pulmonary valve (PV). In (**C**), both the aorta and the main PA are transected. In (**D**), an anastomosis between ascending aorta (with coronary arteries reimplantation) and the native pulmonary valve is shown. (**E**) illustrates flow between RV to the PA (Blue arrow) and a flow between the LV to aorta (red arrow). In (**F**), the Nikaidoh procedure for DORV/TGA with LVOTO is shown. An LVOT is created from LV to aorta (red flow) by harvesting the aortic root from the RV, dividing the hypoplastic native pulmonary valve annulus and the deviated conal septum to the VSD level to relieve the left outflow tract obstruction. The anterior aspect of the LVOT is reconstructed with a VSD patch from the crest of the VSD to the aortic root. RVOT reconstruction (blue arrow) is created by using a pericardial patch. AoV: aortic valve.

**Figure 13:**
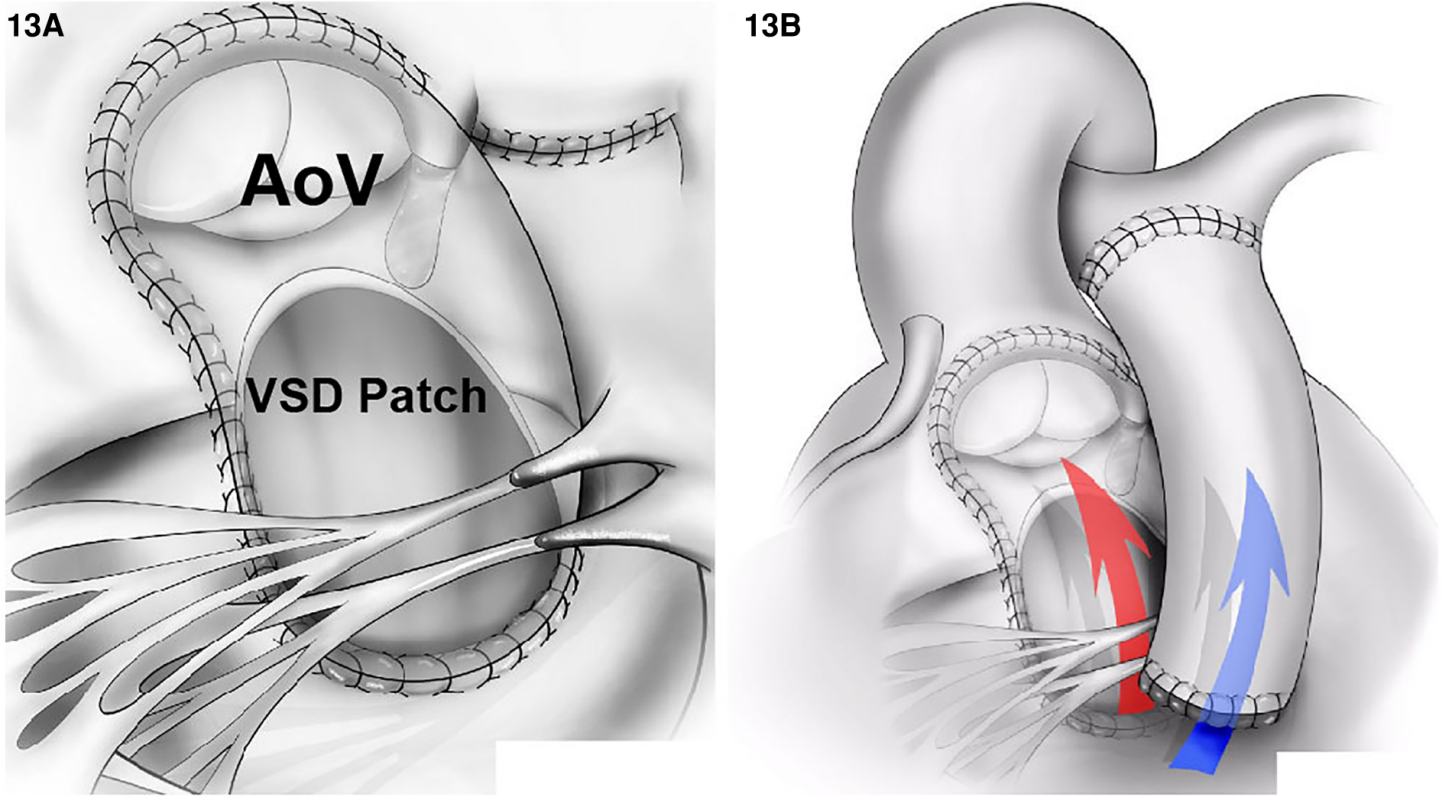
DORV repair using the Rastelli technique. (**A**) An intraventricular tunnel is created between the LV and the aortic valve (AoV) (red arrow in **B**) passing through the enlarged the VSD and resected the conal septum. (**B**) An RVOT reconstruction using a conduit between RV and PA (blue arrow).

In DORV-TGA, the vessel and the ventricular communication are closest to the PA, and this anatomical relationship determines the method of anatomic repair. This subgroup is frequently associated with coronary artery anomalies, aortic arch hypoplasia, and coarctation, thereby increasing the complexity of surgical repair. Different surgical repairs have been used for this subgroup of patients, some of them of historical value in the current times. For example, tunneling the VSD to the pulmonary trunk combined with an atrial switch (Mustard/Senning) is rarely performed in the modern era. Instead, depending on the relationship of the great arteries and the associated cardiac or great vessel anomalies, the following procedures can be performed:

1. The Kawashima procedure: The VSD is baffled to the aortic valve with a patch enlargement of the ventriculotomy. This alternative suits patients with side-by-side great arteries associated with anomalous coronary anatomy that precludes root translocation. Furthermore, a Kawashima procedure can be appropriate for patients when the pulmonary valve is not of adequate size to support the systemic circulation. In this case, the conus should be resected completely to allow rerouting the VSD to the aortic valve (Figure 11). In cases where the great vessel is anterior–posterior relationship related, the distance between the tricuspid valve and pulmonary valve tends to be insufficient for an intraventricular patch. As such, the ventriculotomy patch enlargement is needed to increase the size of the RVOT.
2. Baffle the VSD to the PA combined with an arterial switch operation: Again, this is the most common surgical repair when the pulmonary valve is suitable for systemic circulation since a shorter intraventricular baffle is needed with the subpulmonic VSD (Figure 12). Moreover, whenever there is aortic arch hypoplasia, the aortic repair can be combined with an arch reconstruction. Depending on the great vessel spatial relationship, a LeCompte maneuver may or may not be required. The division of the aorta is especially high in anticipation of the extra length needed for the proximal neo-pulmonary trunk to meet the transverse right pulmonary artery branch. The main pulmonary trunk is transected proximal to the bifurcation.
3. The Rastelli procedure: It is one that includes closing the VSD to the aorta and placing a valved RV to PA conduit for RVOT reconstruction. Alternatively, a direct reconstruction of the RV to PA continuity without conduits after translocation with a LeCompte maneuver (Réparation à l’étage Ventriculaire, REV procedure) can be pursued. These procedures may be preferred when the pulmonary valve is not sufficient to support the systemic circulation due to stenosis or the intraventricular rerouting is toward the aortic valve. In this scenario, the VSD should be enlarged, and the conus resected out routinely to prevent LVOTO and the patch covers both pulmonary and aortic valves to prevent subaortic stenosis. The major difference between a Rastelli and an REV procedure is the material utilized for reconstruction of the RVOT. In the classic description of the Rastelli procedure, a homograft is placed in a heterotopic position to reconstruct the continuity between the RV and the PA (Figure 13). Plication of the proximal pulmonary stump and incorporating the pulmonary valve cusps should be accomplished to avoid blind spaces prone to thrombosis. In the REV procedure, instead of using an RV to PA conduit, the main PA is directly anastomosed to the infundibulum utilizing the LeCompte maneuver and anterior patch enlargement.
4. The Nikaidoh procedure: This procedure entails posterior translocation of the aortic root or double root translocation. A Nikaidoh procedure may result in a more acceptable anatomic repair with better alignment between the LV and the aorta and preservation of the right ventricular volume (Figure 12F). In general, root translocation is considered for all conotruncal anomalies where routing the VSD to the aorta is either not possible or is not desirable. For example, a long adynamic tunnel that compromises native ventricular volume or an LVOT that is not “straight,” resulting in energy loss and with considerable risk of subaortic stenosis at follow-up. Alternatively, in patients with more than moderate right ventricular hypoplasia, a Nikaidoh procedure is preferred as the baffle for the Rastelli may obstruct the RV and pathologically reduce the diastolic volume.

The decision to perform a Nikaidoh procedure or a double root translocation is determined by the suitability of the pulmonary valve to being translocated. Irrespective of this, some anatomic factors we need to address when considering this procedure are

1. size of the pulmonary annulus size,
2. volume of the RV,
3. location of the VSD,
4. abnormal atrioventricular valves attachments or straddling, and
5. coronary anatomy.

In terms of the pulmonary annulus size, we do not offer a Nikaidoh procedure to patients with a pulmonary annulus size of less than 5 mm. This is because in patients with pulmonary annulus <5 mm, the forward movement of the pulmonic root for such a small distance creates angulation when the LVOT is created. This angulation predisposes the patients to LVOTO in the future. In these patients, a Rastelli operation is preferred. Regarding safety of coronary transfer, the only anomaly that excludes patients from undergoing a Nikaidoh is the left anterior descending, right coronary artery (RCA), or a single coronary crossing the RVOT at the level where transection is needed to harvest the root. The Nikaidoh procedure is not recommended if the right coronary artery (RCA) crosses the RVOT but can be performed if the circumflex coronary artery originates from RCA. However, if the circumflex coronary artery is from RCA, this is a relative contraindication to En Bloc rotation of the outflow tracts/double root translocation technique but can be performed successfully in an experienced surgical team (71). Finally, we tend to consider the Nikaidoh procedure in patients with remote VSDs through which the aortic valve cannot be visualized or in the presence of straddling AV valves or abnormal attachments.

The double root translocation was initially described for patients with DORV remote VSD, since the construction of a long prosthetic intracardiac tunnel significantly augments the risk of LVOTO and unfavorable hemodynamics due to the akinetic area. The most favorable anatomy for double root translocation according to Hu et al. (72) is DORV non-committed VSD and DORV-TGA with bilateral conus, with or without pulmonary stenosis.

### Univentricular approach

Contraindications to biventricular repair include severe ventricular hypoplasia, abnormal tricuspid chordae, multiple VSDs, and a straddling mitral valve. This subgroup is the most likely to undergo single ventricle palliation. There are stepwise surgical palliations in the single ventricular pathway. In certain non-committed VSD patients, creation of a non-obstructed intraventricular tunnel may not be felt to be feasible. In other circumstances, there may be severe ventricular hypoplasia, severe atrioventricular valve straddling, or Swiss-cheese VSDs. Thus, the univentricular pathway may be the best management strategy for this subset of patients.

If a single ventricle pathway is elected, significant cyanosis or over-circulation is initially palliated with a systemic to PA shunt (or PDA stent) or PA band placement until 3–6 months of age. Following these first approaches, a bidirectional cavopulmonary connection is created (73). Third stage palliation is completed with a total cavopulmonary connection at 2–3 years of age. It is important that any restrictive VSD be enlarged at or ideally prior to the time of Fontan completion. Although many of these patients require single ventricle approaches, new methods are evolving. For example, a report originating from Japan recently described a successful biventricular repair of DORV with transposition of the great arteries, pulmonary stenosis, and straddling mitral valve (73).

### Post-operative complications and long-term outcomes

The clinical outcomes in patients with DORV have improved significantly over time with the degree of success depending on the complexity of the underlying lesions and the institution’s experience. For example, outcomes among patients with DORV-VSD are excellent. Li et al. (74) published their outcomes, including 129 patients with DORV-VSD, with in-hospital mortality of 1.6%. The surgical outcomes of DORV-Fallot are like those achieved in the TOF, with operative mortality between 0% and 5%. Although classically reported due to the location of the VSD, heart block was only noted in less than 1%. Shim et al. (75) reported their 20-year experience with arterial switch operation for patients with Taussig–Bing anomaly, and there were only 2.2% of patients succumbing to in-hospital death and only one patient (0.7%) undergoing early reoperation due to coronary insufficiency. According to a recent analysis of the STS database by Seese et al. (76) in patients who underwent Nikaidoh, Rastelli, or REV procedure in the US from 2010 to 2019, the operative mortality was 3.1% (95% CI 1.0%–7.0%; *n* = 5) for Rastelli, 4.4% (95% CI 1.4%–9.9%; *n* = 5) for the Nikaidoh, and 11.1% (95% CI 0.3%–48.3%, *n* = 1) for REV patients. A major postoperative morbidity was experienced in 18.8% (95% CI 13.0%–25.7%, *n* = 30) in the Rastelli group, 25.0% (95% CI 17.4%–33.9%; *n* = 29) in the Nikaidoh group, and 22.2% (95% CI 2.8–60.0%; *n* = 2) in the REV group. Unplanned reoperation was reported in 11.9% (95% CI 7.3%–17.9%) among Rastelli/RV to PA conduit placement patients and in 19.8% (95% CI 13%–28.9%) among Nikaidoh patients.

### Late complications

In the Rastelli procedure, the tunnel can be long and can become obstructed over time and the child can also have somatic outgrowth of the conduit. This will ultimately result in the need for RV to PA conduit replacement but can be temporized with catheter-based ballooning and stenting prior to surgery. In these cases of obstruction, there may be RV dysfunction over time. Coronary anomalies increase the risk of unfavorable outcomes in the Nikaidoh procedure.

Kreutzer et al. (77) has reported that the Rastelli procedure can be performed with very low early mortality. However, late mortality occurred in patients with RVOTO and LVOTO. Moreover, the European Congenital Heart Surgeons Association reported a high reoperation rate after the Rastelli procedure for the expected reasons of somatic outgrowth of the conduit over time. From the European Congenital Heart Surgeons Association data, freedom from death, reoperation rate, or reintervention rate of the REV are 90%, 78%, and 64% at 1, 5, and 10 years, respectively (78).

While any of these repairs can result in good outcomes, patient selection and determining the best operation for the patho-anatomy and the patient’s condition are essential. Furthermore, procedural selection also must align with surgeon experience and comfort level or consultation at another institution can be considered if needed.
